# Antioxidant Properties and Reported Ethnomedicinal Use of the Genus *Echium* (Boraginaceae)

**DOI:** 10.3390/antiox9080722

**Published:** 2020-08-09

**Authors:** Ju Jin, Mark Boersch, Akshaya Nagarajan, Andrew K. Davey, Matthew Zunk

**Affiliations:** 1 School of Pharmacy and Pharmacology, Griffith University, Gold Coast Campus, 4222 Queensland, Australia; ju.jin@griffithuni.edu.au (J.J.); akshaya.nagarajan@griffithuni.edu.au (A.N.); mark.boersch@alumni.griffithuni.edu.au (M.B.); a.davey@griffith.edu.au (A.K.D.); 2 Quality Use of Medicines Network, Griffith University, Gold Coast Campus, 4222 Queensland, Australia; 3 Menzies Health Institute Queensland, Griffith University, Gold Coast Campus, 4222 Queensland, Australia

**Keywords:** *Echium* L., phytochemicals, antioxidant activity, traditional uses

## Abstract

The genus *Echium* L. from the Boraginaceae family consists of 67 recognised species. The genus is widely distributed in the Mediterranean, having been documented in the traditional medicine of the area since 300 B.C. Current pharmacological studies have validated early ethnomedicinal properties showing that *Echium* spp. possesses antioxidant, analgesic, anxiolytic, anti-inflammatory, antibacterial, and antiviral effects. Nevertheless, only limited papers report specifically on the phytochemistry of this genus. Furthermore, the potential of utilising extracts from *Echium* species as natural antioxidant preparations has been significantly neglected. For the first time, this review comprehensively describes and discusses the presence of recorded *Echium* species with ethnomedicinal uses, their antioxidative properties in vitro and in vivo when available, and major phytochemical components recognised as potent antioxidants, as well as the possibilities and opportunities for future research.

## 1. Introduction

The genus *Echium* L. (Boraginaceae) consists of 67 recognised species, which are native to North Africa, mainland Europe, and the Macaronesia region (the Azores, Madeira, Canary Islands, and Cape Verde), where they are found to be annual, biennial, or perennial flowering plants [1]. Interestingly, 70% of species endemic to the Macaronesian archipelagos are found to be woody shrubs [2], while continental species are mostly herbaceous. Numerous species belonging to *Echium* L. have been introduced into North America and Australia as ornamental and garden plants [3].

The ethnomedicinal history of the *Echium* species can be traced back to 300 B.C. in the Mediterranean area [4]. Reports indicate various species have been used as folk medicine in the region, utilised predominately for their sedative, anti-inflammatory, antioxidant, and anxiolytic properties, treating ailments including fissures of the hands, general abrasions, and even snakebites [5,6,7]. Due to extensive colonisation for the past 500 years, the *Echium* species have been introduced and distributed to many countries worldwide [8]. In general, they are hardy plants that adapt well to harsh environments. Due to their ability to grow and thrive in austere environments, they have particularly thrived in Australia, South Africa, and America, out-competing native plant species and, hence, are considered invasive [9,10,11].

In regards to their phytochemistry, a variety of the biologically active constituents have been isolated from *Echium* species, such as naphthoquinones, flavonoids, terpenoids, and phenols [12], which exert anxiolytic [5,6,13,14], antioxidant [15], anti-inflammatory [16], antibacterial [17], and antiviral [18] effects. Although there have been reviews conducted on the distribution of *Echium* species in certain areas [2,19], as well as some reports on the pharmacological properties of *Echium amoenum* in particular [20,21], there is currently no comprehensive review concerning the *Echium* genus as a whole. To date, the promising benefits and medicinal use of the *Echium* genus have been largely neglected. This review will focus on the antioxidant activity of *Echium* spp., which are documented to be valuable in a variety of pathological conditions and disease processes.

### Aim and Methodology

This narrative literature review aims to consolidate the literature to date in English and, therefore, represents a significant approach towards the ethnomedicinal knowledge regarding traditional use and, in particular, antioxidative activity of preparations and isolated pure compounds from the 67 accepted species of the *Echium* genus.

Methodology: Regarding the methods used in obtaining the literature to be reviewed, time of publication was not an exclusion criterion. One exclusion criterion existed when choosing literature to review. That was, all reports had to be published in the English language. The authors accept that this may limit some literature from more “traditional sources”, particularly from uses around the native regions for *Echium* spp. However, all reports searchable under the keywords, including MESH terms in common databases, such as Embase and Medline, were included without alteration. Therefore, this review is an accurate representation of the current therapeutic knowledge for the use of *Echium* spp.

## 2. Ethnomedicinal Uses of *Echium* Species

Species of *Echium* have been used for thousands of years in the Mediterranean area as folk medicines possessing depurative, diaphoretic, diuretic, and mood-enhancing properties [5,12,22]. 

*E. amoenum* is the one species in this genus most-studied to date. It is a wild annual herb, found to have medicinal benefits utilised by the Romans in the early third century BC. Brewing or boiling of this species in water followed by ingestion was a traditional way to treat a cold or fever. Alternatively, mixing the plant leaves with wine was believed to have had a mood-enhancing effect, and this positive effect on mood was also noted by the Greek poet Homer [4]. In Iran, petals of this plant (locally known as ‘*Gol-e-Gavzaban*’ or ‘*Lesan-al-sou*’), when prepared via decoction and mixed with honey before being taken orally, produce both anxiolytic and sedative effects. Furthermore, this particular species was also consumed orally in Iran to relieve minor ailments, such as a sore throat, cough, or flu-like symptoms [4,23,24]., which correlates with the early Roman use. In other parts of the world, such as Turkey, France, and Italy, the roots and aerial part of *E. amoenum* were used for the treatment of mouth ulcers and respiratory infections, being reported to exhibit depurative, diaphoretic, diuretic, and emollient effects [17,25,26].

Another species commonly used in folk medicine in the Mediterranean area is *Echium vulgare* [27,28], commonly known as Viper’s Bugloss or Blueweed in English (‘*Havaciva’* in Turkish ethnobotany). The root of *E. vulgare* has been used to help improve wound healing, bruising, pulled muscles, ligaments, and sprains in both Turkey and Germany [27,28,29,30]. In Turkey, an ointment was prepared as a mixture of the cooked root with butter, which was then applied to these injuries topically [27]. Additionally, the aerial anatomy, leaves, and flowers can be used medicinally as a diuretic and cough medicine [31]. Furthermore, *E. vulgare* has been utilised as a remedy for viper snake bites and scorpion stings [32,33,34]. Unfortunately, the details for treatment of snake bites and cough is poorly documented, with no detail of preparations available. 

*Echium italicum* is another popular folk medicine in Turkey (known as ‘*kuşkonmanz*’ and ‘*dikeni*’, for the herbs and leaves, respectively), being used to improve wound healing, blisters, and bruises. In eastern Turkey, the crushed leaves were utilised as a rub to treat abscesses and rheumatic pain. Haemorrhages were often treated with a mixture of herbs and flour, aiding the blood to clot [35]. An ointment made by roasting *E. italicum* root with butter was used after an injury to aid wound healing [27,28,36]. Furthermore, in Italy, it is recorded that a decoction of this plant was used for its depurative, diaphoretic, diuretic, and even emollient properties. Interestingly, this species is reported to have been used primarily to improve respiratory infections [26], however the preparation and how it was administered for this purpose is not clear.

In addition to the three species mentioned above, the root of *E. angustifolium* and the herbs of *E. parviflorum, E. plantagineum*, and *E. russicum* have also been reportedly used in Turkish traditional medicine [27,28,37,38,39]. For example, the aerial parts of *E. plantagineum* are locally known as ‘*Engerek ut’*, and its decoction is prepared as a tea with diaphoretic and diuretic effects [27] (Table 1).

More species have been reported as possessing pharmacological activity in the Middle East. A report from Saudi Arabia describes *E. arabicum* being found to exert antiplasmodial and antitrypanosomal activity [40]. In Jordan, *E. glomeratum* (locally known, ‘*Sag Al-hamam’*) is said to possess analgesic, diaphoretic, and aphrodisiac effects and has been used for the treatment of snake bites. *E. judaeum* (‘*Lesan Al-Thoor*’) has also been used in the Jordanian provinces for its sedative effects, managing hyperactivity, anxiety, and improving certain dermatological conditions [41].

In Spain, the root of *E. flavum* Desf. was mashed or fried in olive oil to make up a red-coloured liquid to externally treat ulcers and herpes wounds [42]. More recently, this reddish colour was confirmed to be caused by the concentration of naphthoquinones, which are known to have a variety of biological activities [11]. In the island of Cape Verde, *E. stenosiphon* and *E. vulcanorum* were known as ‘*língua-de-vaca’* (cow tongue), being used to treat coughs and gastrointestinal diseases [43]. Additionally, the seed oil of *E. vulcanorum* is said to have been used as a potential dietetic supplement [44], however the specific therapeutic activity of *E. vulcanorum* in this context has not been clearly explained in the literature.

In summary, thirteen *Echium* species have been used as folk medicines across the endemic geographical distribution of Asia, Europe, and Macaronesia archipelagos. Among the plant parts, the roots are the most-used, followed by the aerial parts. *Echium* species exert sedative, anti-inflammatory, antioxidant, and anxiolytic pharmacological properties. As a result, these species are largely used to treat respiratory problems, ulcers, mental health ailments, and aid in wound healing (Table 1).

## 3. Recorded Antioxidant Activity of the *Echium* Genus

### 3.1. Antioxidants

Various alcoholic extracts of the petals of *Echium* have demonstrated different antioxidant activity levels [15,49,50,51,52]. *E. amoenum* is one of the most studied species in the genus for antioxidant activity. Asghari et al. [52] reported that a decoction and hydroalcoholic extracts of *E. amoenum* showed more promising antioxidant activity than those extracted with methanol alone. This was evaluated by an OH radical assay, α,α-diphenyl-β-picrylhydrazyl (DPPH), 2,2’-azino-bis(3-ethylbenzothiazoline-6-sulfonic acid (ABTS), and the ferric reducing power assays, which are all common assays for determining total antioxidative potential, measuring parameters, such as free radical scavenging outcomes. Furthermore, the hydroalcoholic extracts showed the highest radical scavenging effects (IC_50_ 110.8 μg/mL) in OH radical assays. The highest DPPH and ABST results were shown from the decoction extract with IC_50_ values of 22.8 μg/mL and 17.1 μg/mL, respectively [52].

Shirin Adel Pilerood and Jamuna Prakash [50] reported in their study that a water extract of *E. amoenum* showed the highest antioxidant properties in a DPPH assay, whereas the acetone extracts showed the least activity. Petals of this species exhibited higher total phenolic content (TPC) (1540 mg/100 g), total flavonoid content (TFC) (4.54 ± 0.042 mg), and tannin content (2.47 ± 0.064 mg) when extracted by hot water. Overall, the antioxidant properties of *E. amoenum* could explain many of the biologically significant outcomes as recorded in the ethnomedicinal record. Moreover, this tradition of using tea (particularly in Iran) as a vehicle could be clinically significant, as water-based formulations are biologically applicable compared to organic solvents as formulations. However, it is always important to remember that water stable and soluble phytochemicals are not always bioavailable [53], and therefore hot water extracted phytochemicals from *E. amoenum* require further pharmacological investigation.

Importantly, many reports exist regarding the evaluation of antioxidant activity for species in the genus, not recorded in the English ethnomedicinal literature. The antioxidant activity of hydromethanolic root extracts of *E. pycnanthum* (a subspecies of *E. humile* Desf.) collected in southern Algeria was reported on by Chaouche et al. [54]. The results obtained using DPPH, iron-reducing power, ABST, iron chelation, and β-carotene assays showed a high flavonoid content of 16.26 ± 1.4 mg of CE/g DW. *E. sericeum (E. creticum),* a species found in Egypt, also exhibited antioxidant effects in a DPPH assay [55]. This was speculated to, likewise, be due to the presence of the flavonoids in the extract. *E. rauwolfii* was found to have the highest scavenging activity at IC_50_ = 14.3 μg when extracted with butanol, whilst the ethyl acetate extract presented the weakest activity at IC_50_ = 432.3 μg [56]. Sarra Kefi et al. [57] indicated that the ethyl acetate extracts of the aerial parts of *E. arenarium* grown in Tunisia were characterised by a high antioxidant activity with the highest TPC compared to those obtained from hydroethanolic, aqueous, and cyclohexane extracts. These extracts consequently showed the best DPPH inhibition (IC_50_ = 1.1 μg/mL) and β-carotene bleaching inhibition (IC_50_ = 9.94 μg/mL).

In Serbia, six antioxidant activity assays, including metal-chelating (Fe^2+^), FRAP, phosphomolybdenum method, OH radical, DPPH, and ABTS radical scavenging activity were carried out on *E. vulgare* and *E. rubrum.* It was subsequently determined that *E. vulgare* contains higher antioxidant activity, including higher TPC and TFC values [58]. Again, this outcome could explain the rich ethnomedicinal use of *E. vulgare* in the Mediterranean area for centuries.

Interestingly, natural antioxidant activity is influenced by the drying process and extraction methods. Fatemeh Nadi et al. [59] demonstrated that at an air velocity of 1 m/s at 50 °C, the dried petals showed the highest DPPH values (61.16%). Yet the optimal drying condition was found to be 60 °C and an air velocity of 0.86 m/s, which obtains the optimum level of TPC, TFC, anthocyanin content, and antioxidant capacity. Additionally, microwave-assisted extraction (MAE) of polysaccharides from *E. vulgare* flowers showed a moderately higher scavenging hydroxyl radical ability and significant DPPH values (89.3%) compared to the control (78.6%), which relates to a higher antioxidant activity [60]. Furthermore, growing in metallicolous and nonmetallicolous soil environments influenced the antioxidant activity of *E. vulgare.* Plants that were exposed to short-term zinc stress generally showed higher antioxidant activity, as well as total phenolics and flavonoids [61].

Hashemi et al. [62] evaluated the sun protection factor (SPF), antioxidant capacity, as well as flavonoid and phenol contents of methanolic extracts of petals of *E. amoenum.* The extraction was achieved using three different methods (percolation, Soxhlet, and ultrasonically). The phenol and flavonoid contents and antioxidant activity of these extracts were evaluated using the Folin–Ciocalteu reagent, aluminium chloride methods, and DPPH assays. The results showed that with the assistance of percolation, the petal extracts showed the highest antioxidant activity (IC_50_ = 162.3 ± 4.1 μg/mL) and highest flavonoid contents (42.64 ± 1.6 QE/g) but the lowest SPF value (0.124 ± 0.00 at 2 mg/mL). There was no correlation between SPF and the content of phenols, flavonoids, or antioxidant properties in *E. amoenum*. Interestingly, although this correlation is absent in *E. amoenum*, Hashemi et al. reports a significant correlation between SPF and phenolic and flavonoid content in other plant species.

Comparisons of the antioxidant properties of *Echium* species were investigated by Sakineh Abbaszadeh [63] who found that hydroalcoholic extracts of *E. amoenum* seeds yielded potent antioxidant properties. These extracts yielded the highest DPPH and FRAP values when compared to the leaf and stem extract from *E. amoenum*, as well as the same extract from *E. italicum*. Leaves of *E. amoenum* showed the highest TPC values (119.50 ± 2.00 mg GAE/g DW), with the seed extract showing the highest TFC values (62.17 ± 3.59 mg QE/g DW). Moreover, Nuraniye and Eruygur [29] compared the antioxidant activity of *E. italicum* L., *E. vulgare* L., *E. parviflorum*, and *E. angustifolium* using DPPH assays and by comparing their ferrous ion chelating ability. Generally, root extracts were attributed to having better antioxidant effects than the herb extracts. This is hypothesised to be due to the roots being rich in phenols, such as tannins, anthraquinones, terpenoids, and flavonoids [64]. A higher DPPH scavenging activity (81.43 ± 0.01%) was achieved by the ethanolic root extract of *E. italicum*, whilst a higher iron-chelating activity (48.69 ± 0.04%) was achieved by *E. parviflorum* ethanolic root extracts. During the investigation of the TPC and TFC quantities of these four species, it was shown that the root of *E. angustifolium* obtained the highest TPC and TFC values at 38.86 ± 0.008 mg GAE/g and 56.12 ± 0.01 mg QE/g, respectively. This was followed by the *E. italicum*, *E. vulgare*, and *E. parviflorum* extracts.

The results outlined regarding *E. angustifolium* having the highest TPC and TFC quantities were not supported by the work of Vukajlović et al. [65], who was able to show that *E. vulgare* presented the highest total antioxidative capacity (TAC) followed by *E. italicum* and *E. rubrum.* Although *E. rubrum* showed the least TAC in these investigations, this species had more significant results in the DPPH, FRAP, and reducing power assays with 78.18 ± 8.73 mg TE/g DW, 122.07 ± 11.30 mg TE/g DW and 638.74 ± 65.40 mg BHTE/g, respectively. There is disagreement within the literature regarding which species has the more antioxidative capacity and presents an opportunity for further investigation.

In general, assays, such as DPPH, ABTS, and the ferric reducing power assay (FRAP), were conducted to evaluate the antioxidant capacity for extracts of numerous *Echium* species. Plants were subjected to different solvent (hydroalcoholic, aqueous, cyclohexane, ethyl acetate, hexane) to obtain extracts. Hydroalcoholic extracts commonly showed the highest antioxidant activity in all investigations, whilst ethyl acetate showed the least. It was also seen that in general the root extracts of the genus showed higher antioxidant activities than other parts of the plant. The results showed this antioxidant capacity might be due to the presence of phenolic compounds. This high level of phenolic compounds could be the result of using hydroalcoholic solutions as an extraction solvent and indicates that the roots of the plants contain the highest concentrations of phenols and other chemicals associated with antioxidant properties. Among all the *Echium* species, *E. amoenum* is the most studied, with the percolation extraction technique consistently obtaining the highest antioxidant activities.

### 3.2. Oxidative Stress

When free radicals accumulate within a cell and overpower the buffering effects of oxidants, oxidative stress is the result. Oxidative stress has been established as a major contributor to many pathological conditions and diseases [66]. By using the antioxidant effects of plants, some disease states can be mitigated. *E. amoenum* is the most studied species related to oxidative stress. Its petal*s* have been investigated in vitro by evaluating the oxidative stress on human vascular endothelial cells (HUVECs) [67]. Pretreatment with petal extracts on H_2_O_2_-induced oxidative stress HUVECs cell models reduced the rate of cell death. In this case, a reduction of hydroperoxide’s concentration, assessed by FOX-1 assays, and an increasing value of ferric reducing antioxidant power (FRAP) in both intra and extracellular fluid were observed. This is further confirmed by in vivo studies by Noroozpour et al., which showed that the hydroalcoholic petal extract of *E. amoenum* had a protective effect on selenite-induced cataracts in rats [51]. Likewise, Abbasi Larki et al. [68] demonstrated that the petal of *E. amoenum* had a protective effect on permethrin-induced rat models. The pretreatment with this extract showed a greatly reduced inflammatory response of cerulein-induced acute pancreatitis in mice [69]. Mahsa Kamali et al. [70] reported that *E. amoenum* could reduce the rate of teratogenicity induced by the antiepileptic medication lamotrigine in mice. Moreover, a clinical trial of 38 volunteers was carried out to investigate the antioxidant properties of *E. amoenum* [71]. All the volunteers were administered 7 mg/kg of *E. amoenum* flower decoction twice every day for two weeks. The results showed that after 14 days of oral consumption of the flower decoction of *E. amoenum*, blood lipid peroxidation levels were reduced (from 24.65 ± 11.33 to 19.05 ± 9.7 nmol/mL), and total antioxidant capacity of blood (from 1.46 ± 0.51 to 1.70 ± 0.36 μmol/mL), as well as total thiol molecules in the blood (from 0.49 ± 0.11 to 0.56 ± 0.12 μmol/mL), was increased [71]. These findings suggested that *E. amoenum* could ameliorate the pathological conditions resulted from oxidative stress and could be used as a nutritional supplement.

### 3.3. Phytochemicals from Echium spp. That Possess Antioxidant Activity

Table 2 summarises to date the bioactive phytochemicals that have antioxidant capacity identified from *Echium* spp. (refer to Figure S1 for chemical structures). Research on *Echium* phytochemistry so far has revealed the existence of phenolic acids, flavonoids, tannins, lignans, and naphthoquinones isolated from the aerial plant, shoots, and roots of the plant. These types of compounds could certainly account for the promising antioxidant activity found in some of the traditional preparations, as documented in Section 2. One of the most bioactive classes of compounds that are responsible for antioxidative properties is phenolic compounds [58,63,64]. These secondary metabolites contain one or more hydroxylated aromatic rings commonly occurring in plants and include compound families, such as phenolic acids, flavonoids, tannins, lignans, and quinones. Structurally, the number of hydroxyl moieties bonded to the ring systems, and where they are located, is related to the antioxidative effect [72]. This is due to the hydrogen donating properties of phenols subsequently quenching free radical-induced lipid peroxidation and enhancing the ability to scavenge free radicals [72,73]. Hence, there has been increased research interest in phenolic compounds, as they are effective in treating pathological conditions and diseases related to oxidative stress.

Numerous reports have looked at the determination of the presence of phenolics in specific species in the genus *Echium*. Saponins and tannins were found within the aerial organs of *E. italicum* by Fazly Bazzaz et al. [74]. Similarly, Chaouche, T. [75] revealed that phenols (flavonoids and tannins), as well as steroids (saponins, sterols, and triterpenes) exist in both the leaves and roots of *E. pycnanthum.* Furthermore, the petals of *E. amoenum* contained anthocyanidin (13% *w/v*), flavonoid aglycons (0.15% *w/v*), volatile oil (0.05% *w/v*) with δ-cadinene (24.25% *w/v*) [24], which certainly could account for the vast ethnomedicinal uses of this particular species. Sousa et al. [76] evaluated the antioxidant capacity of bee pollen extracts, in particular flavonols and anthocyanins extracted from *E. plantagineum.* In this study, oxidative stress models were conducted in vitro (Caco-2 cells induced by *tert*-butyl hydroperoxide (*t*-BHP)). Interestingly, the isolated anthocyanins moderately impeded antioxidant potential, while the isolated flavonols partly protected the Caco-2 cells under oxidative stress.

Flavonoids are commonly found in nature and are well known to contribute to the various aromas and colours of plants [77]. Likewise, in the *Echium* species, flavonoids together with glycosylation and conjugation (especially C3 substituted flavonoids) have been extensively identified. This includes flavones, such as apigenin and luteolin, flavonols, such as quercetin and kaempferol, as well as anthocyanins, such as delphinidin and cyanidin (Table 2 and Figure S1). Identification of flavonoids within *Echium* has been predominately conducted by comparison of analytical standards via high-performance liquid chromatography (HPLC) attached to various analysis detectors, such as a diode-array detector (DAD) or pulsed amperometric detector (PAD). Kefi et al. [57] reported that four flavonoids (luteolin-7-*O*-glucoside, myricitrin, myricetin, and quercetin) were identified from *E. arenarium* extracts by RP-HPLC analysis, with Luteolin-7-*O*-glucoside (60.56 μg/mg DE) the major flavonoid among all the compounds. Identification of nineteen flavonoids, in various species, both in coloured and noncoloured extracts have been described, including kaempferol, peonidin, cyanidin, malvidin, and their monoglucoside or disaccharide C3-linked derivatives, with kaempferol-3-*O*-neohesperidoside found to be the major constituent [78,79,80]. Radwan et al. [55] used proton nuclear magnetic resonance (^1^H-NMR) and fast atom bombardment (FAB-MS) to characterise four flavonoids, such as apigenin, luteolin-7-*O*-rutinoside, apigenin-7-*O*-rhamnoside, and quercetin-3-*O*-rhamnoside. These compounds were identified in *E. sericeum (E. creticum*).

Phenolic acids are also commonly distributed in *Echium* species. One of the best-characterised compounds isolated from the *Echium* genus is rosmarinic acid, which is found in several *Echium* species, such as *E. amoenum*, *E. russicum*, and *E. vulgare* [24,38,90,94]. Moreover, when extracted with hot water, *E. amoenum* petals were shown to have the highest content of rosmarinic acid within these species [52]. Other phenolic acids have been well characterised in *E. russicum,* including salvianolic acid A, rabdosiin, lithospermic acid, and eritrichin (globoidnan A) [38]. All of these phenolic phytochemicals could explain the ethnomedicinal success of these species and represent a potential source of potent antioxidants, antivirals, anti-inflammatories, and potentially, antibacterial agents, which have limited published biologically related mechanistic data to date.

Another identified class of compounds that have shown significant antioxidant capacity is naphthoquinones, represented by shikonin and its derivatives. Papageorgiou et al. [95] has comprehensively reviewed shikonin and its enantiomer alkannin from the historical aspect as well as its chemical and pharmacological properties. Furthermore, Chen et al. [96] has reported on the cellular mechanistic activity of these compounds. Shikonin and its esterified derivatives were mainly found in the bright red root periderm of several *Echium* species [24,81,82,84,86], which correlates well with the reddish ointment used as folk medicine. It has also been shown that shikonin derivatives are present within the callus culture of *E. italicum* and *E. plantagineum* [85,88] (Table 2, Figure S1). Durán et al. [81] showed that *E. gaditanum* contained the highest concentration of 3,3-dimethylacrylshikonin, interestingly independent of their collection locations. As for *E. plantagineum*, the compounds with the highest concentrations within the extracts were found to be 3,3-dimethylacrylshikonin and acetylshikonin [86]. In another study, Weston et al. [11] showed higher concentrations of naphthoquinones were present in the lower geo-elevations than those isolated from plants that are collected from cooler and higher elevations. The colour of the extracts may be indicative of their contents, with bright red colours attributed to higher levels of shikonin, deoxyshikonin, and acetylshikonin [86].

Additionally, a limited number of steroids have been isolated from *Echium* to date. Stigmast-4-ene-3,6-dione and β-sitosterol were identified in *E. vulgare*. β-sitosterol is reported to have a significant antioxidant capacity [97,98]. It is likely more steroids of this type will be identified in other species with time, as phytochemicals appear to be highly conserved within this genus.

In summary, a variety of pure compounds that may account for the antioxidant properties of *Echium* have been isolated. Apart from polyphenols, the presence of naphthoquinones might also play an important part in the antioxidant capacities of plant extracts. However, these classes of compounds have been rarely characterised in the *Echium* species. No specific compounds elucidated from the classes of saponins and unsaturated terpenoids have been investigated thus far. The literature to date identifies that there is extensive future work to be carried out in the isolation and characterisation of biologically active phytochemicals from *Echium* spp. This future work will undoubtedly link the past ethnomedicinal use to new phytochemical classes of biological significance.

## 4. Conclusions

Plant-based extracts and phytochemicals have played a major role in treating human diseases. Despite currently being used as ornamental and garden plants, numerous *Echium* species have been used for millennia as folk medicine in the Mediterranean area. Petals, leaves, roots, and aerial parts have been used for a wide range of diseases, such as respiratory problems, ulcers, mental health-related issues, and wound healing. Recent studies have shown that both crude extracts and isolated compounds of *Echium* species exhibit promising pharmacological activities, with bioactive compounds, such as flavonoids, terpenoids, saponin, phenolic acids, and naphthoquinones, present. These bioactive compounds, either used by themselves or as mixtures, correlate well with the reported traditional ethnomedicinal uses.

Importantly, the genus *Echium,* based on the various ethnomedicinal uses reported, would be expected to produce promising bioactive constituents. However, we note that the current studies published are limited by reliable characterisation and identification of the isolated classes of phytochemicals as well as investigations on their biological activity at the molecular level, which includes mechanistic studies.

This review has identified and discussed potential antioxidants that the various *Echium* species possess. Moreover, the reported ethnomedicinal uses (available in English) have been outlined and correlated this with modern antioxidant research for the first time, which is convincingly connected by isolation of therapeutically relevant phytochemicals. Furthermore, for the first time, this review has listed all of the phytochemicals with antioxidant activity isolated from *Echium* and discussed many of the more promising compounds concerning ethnomedicinal uses and future research. What is apparent from the literature is that further investigation into compound isolation and elucidation is required for more than half of the recognised species. Moreover, in vitro and in vivo biological antioxidative determination and evaluation is also missing from the literature for most species, as well as further categorisation of the mechanism of antioxidant action. The gaps that we have identified will allow exploitation of the antioxidant properties of this genus for formulation into the potential treatments of a wide range of common illnesses and disease states and subsequently, will lead to the significant therapeutic application of preparation based on *Echium* in the future.

## Figures and Tables

**Table 1:** Reported ethnomedicinal * uses of *Echium* spp.

Species	Local Name	Part	Uses	Country
*E. amoenum* Fish. and C.A. Mey.	*Gol-e-Gavzaban/Lesan* *-* *al* *-* *sou*	Petals	Demulcent, anti-inflammatory, and analgesic, especially for common cold, pneumonia, anxiolytic, sedative, and other psychiatric symptoms, including obsession [4,23,24,25]	Iran
		Flower and leaves	Antifebrile, antidepressant, circulatory heart diseases, pulmonary complaints, inflammatory swellings, laxative, emollient [17]	France
		Plant	Heart palpitation [25]	Iran
		Root	Ulcers [25]	Turkey
	*Viperina*	Aerial part	Depurative, diaphoretic, diuretic, healing respiratory infections [26]	Italy
*E. angustifolium* Miller.	*Kızılcık dikeni, Engerek otu*	Roots	Wound healing, ulcer [29]	Turkey
*E. arabicum* R. Mill.			Antiplasmodial and antitrypanosomal activity [27,40]	Saudi Arabia
*E. flavum* Desf	*Ra* *í* *z colorá*	Root	Antiseptics and wound healing for ulcers and herpes [42]	Spain
*E. glomeratum* Poir.	*Sag Al-hamam*		Analgesic, diaphoretic, aphrodisiac, snake bites [37]	Jordan
*E. hypertropicum* Webb.	*língua-de-vaca*	Seed oil	Cough and gastrointestinal diseases [44,45]	Cape Verde
*E. italicum* L.	*Kuşkonmanz/Viperina*	Aerial parts	Wound healing, diaphoretic, emollient, diuretic [26,27,35]	Turkey, Italy
	*Dikeni*	Leaves	Rheumatic pain, bruises and blisters [35]	Turkey
		Roots	Wound healing, ulcer, rheumatic pain, blister, treat bruises [36]	
*E. judaeum* Lacaita	*Lesan Al-Thoor*		Hyperactivity, nervousness, general weakness, eczema and dermatological ailments, analgesic, aphrodisiac, diaphoretic [41]	Jordan
*E. parviflorum* Moench	*Kızıl Engerek otu*	Aerial parts [27]		Turkey
*E. plantagineum* L.	*Engerek otu*	Aerial parts	Diaphoretic and diuretic [46]	Turkey
			Cough and wound healing [47]	Spain
*E. russicum* S. G. Gmel.	*Red burgloss*		Viper bites [38]	
	*Havaciva*	Root/Vulnerary	Fissures on hand, wound healing [28,39]	Turkey
*E. stenosiphon* Webb.	*língua-de-vaca (cow tongue)*		Cough syrup, treatment of cough and gastrointestinal diseases [44,45]	Cape Verde
*E. vulcanorum* A. Chev.	*língua-de-vaca (cow tongue)*	Seed oil	Dietetic [44]	Cape Verde
*E. vulgare* L.	*Havaciva*	Roots	Wound healing, ulcer [27,28,29], bruising, pulled muscles, ligaments and sprains [30]	Turkey, Germany
	*Havaciva* *Cua de porc*	Aerial parts	Diuretic [27,29], snakebites [33,34]	Turkey Spain
		Not described	Cough [31]	Spain
	*Viper’s bugloss*	Leaves, flower	Diuretic [48], bruises and sprains	Germany

* Ethnomedicinal uses in Table 1 have been reported as recorded in the literature. These include uses for human diseases only. There has been no attempt to further interpret this information within this Table, and therefore the information presented is a true representation of what has been published in English to date.

**Table 2:** Pure phytochemicals isolated from *Echium* spp. that have reported antioxidant activity.

Plant Name	Plant Part	Constituents Class	Constituents	Analysis Methods
*E. arenarium (Guss)*	Aerial parts [57]	Flavonoids	Luteolin-7-*O*-glucosides [57]	RP-HPLC [57]
			Myricetin [57]	
			Quercetin [57]	
			Myricitrin [57]	
	Callus [24]	Phenolic acids	Rosmarinic acid [24]	TLC, HPLC, UV, IR, ^1^H-NMR, ^13^C-NMR [81]
*E. angustifolium*	Root [82]	Naphthoquinones	Shikonin [82]	HPLC-UV [82]
			Acetylshikonin [82]	
			Deoxyshikonin [82]	
			3,3-dimethylacrylshikonin [82]	
			2-methyl-*n*-butyrylshikonin [82]	
			Isovalerylshikonin [82]	
*E. gaditanum*	Root periderm [81]		Shikonin [81]	LC-MS/MS [81]
			Acetylshikonin [81]	
			Deoxyshikonin [81]	
			3,3-dimethylacrylshikonin [81]	
*E. italicum*	Shoots, root [83]	Phenolic acids	4-hydroxybenzoic acid [83]	HPCE [83]
			Hydrocaffeic acid [83]	
			Rosmarinic acid [83]	
	Root [27,82,83,84]	Naphthoquinones	Acetylshikonin [27,82,84]	HPLC-UV [82,84]; HPCE [83]; TLC, ^1^H-NMR, ^13^C-NMR [27]; HPLC-VIS,HPLC-MS, ^1^H-NMR, ^13^C-NMR [84]
			Angelylshikonin [84]	
			Deoxyshikonin [27,84]	
			Isobutyrylshikonin [84]	
			Isovalerylshikonin [27,82,84]	
			Propionylshikonin [84]	
			Shikonin [27,83,84]	
			Tiglylshikonin [84]	
			2-methyl-*n*-butyrylshikonin [27,82,84]	
			3,3-dimethylacrylshikonin [82]	
	Callus [85]	Naphthoquinones	Shikonin [85]	TLC, HPLC, preparative HPLC, UV, ^1^H and ^13^C-NMR [85];
*E. judaeum*	Aerial part [41]	Flavonoids	Kaempferol [41]	UPLC-MS [41]
			Luteolin [41]	
	Aerial part [41]	Phenolic acids	Rosmarinic acid [41]	UPLC-MS [41]
	Aerial part [41]	Coumarin	Aesculin [41]	UPLC-MS [41]
*E. parviflorum*	Root [82]	Naphthoquinones	Acetylshikonin [82,86]	HPLC-UV [82]
			Deoxyshikonin [82,86]	
			Isovalerylshikonin [82]	
			Shikonin [82,86]	
			2-methyl-*n*-butyrylshikonin [82]	
*E. plantagineum*	Bee pollen [78,79,80]	Flavonoids	Cyanidin [78]	HPLC-DAD [76,79]; HPLC-PAD-MS; DAD, ESI-MS [78];
			Cyanidin-3-(6″-malonylglucoside) [78]	
			Delphinidin [76,78]	
			Isorhamnetin-3-*O*-rutinoside [80]	
			Kaempferol-3-*O*-glucoside [76,79,80]	
			Kaempferol-3-*O*-neohesperidoside [79,80]	
			Kaempferol-3-*O*-neohesperidoside-7-*O*-rhamnoside [76,80]	
			Kaempferol-3-*O*-(4′-rhamnosyl) neohesperidoside [76,79,80]	
			Kaempferol-3-*O*-(3″/4″acetyl)-neohesperidoside isomer [76,79,80]	
			Kaempferol-3-*O*-rutinoside [76,79,80]	
			Kaempferol-3-*O*-sophoroside [76,79,80]	
			Malvidin-3-*O*-rutinoside [76,78]	
			Peonidin [78]	
			Petunidin-3-*O*-glucoside [76,78]	
			Petunidin-3-*O*-rutinoside [78]	
			Quercetin-3-*O*-sophoroside [80]	
			Quercetin-3-*O*-neohesperido-side [76,80]	
	Roots and rhizosphere [11,81]	Naphthoquinones	Deoxyshikonin [11,81,87]	LC-ESI/MS [86];LC-MS/MS [81]; UHPLC/Q-ToF MS [11]
			Isobutyrylshikonin [87]	
			Isovalerylshikonin [87]	
			Shikonin [11,81,87]	
			Acetylshikonin [11,81]	
			3,3-dimethylacrylshikonin [11,81,87]	
			2-methyl-*n*-butyrylshikonin [87]	
			3-hydroxyisovalerylshikonin [87]	
			Propionylshikonin [87]	
	Callus [88]	Naphthoquinones	Acetylshikonin [88]	TLC, UV, IR, ^1^H-NMR, CD [88];PLC-MS-DAD-QToF [87]
			Isobutyrylshikonin [88]	
			Isovalerylshikonin [88]	
			3-hydroxyisovalerylshikonin [88]	
			3,3-dimethylacrylshikonin [88]	
*E. pycnanthum* POMEL	Root [89]	Naphthoquinones	Acetylshikonin [89]	^1^H and ^13^C-NMR [89]
			Angelylshikonin [89]	
			Isobutyrylshikonin [89]	
			Isovalerylshikonin [89]	
			3,3-dimethylacrylshikonin [89]	
			2-methyl-*n*-butyrylshikonin [89]	
			Propionylshikonin [89]	
			Shikonin [89]	
			Tiglylshikonin [89]	
*E. russicum*	Shoots, root [83]	Flavonoids	Rutin [83]	HPCE [83]
	Shoots [83,90]; Root [38,90]	Phenolic acids	Eritrichin (globoidnan A) [38]	CC, UV, IR and NMR [38]; CZE [90]; HPCE [83]
			Lithospermic acid [38]	
			Rosmarinic acid [83,90]	
			Rabdosiin [38]	
			Salvianolic acid A [38]	
	Root [38,83,90]	Naphthoquinones	Acetylshikonin [38]	CC, UV, IR, MS and NMR [38]; CZE [90], HPCE [83];
			Angeloylshikonin [38]	
			deoxyshikonin [38]	
			Isobutylshikonin [38]	
			Isovalerylshikonin [38]	
			Shikonin [83,90]	
			Tigloylshikonin [38]	
			3-acetoxyisovalerylshikonin [38]	
			3-hydroxyisovalerylshikonin [38]	
			3,3-dimethylacrylshikonin [38]	
*E. sericeum (Vahl)*		Flavonoids	Apigenin [55]	TLC, HPLC, UV, ^1^H-NMR and FAB-MS [55].
			Apigenin-7-*O*-rhamnoside [55]	
			Luteolin-7-*O*-rutinoside [55]	
			Quercetin-3-*O*-rhamnoside [55]	
*E. vulgare*	Aerial part [91]	Phenolic acids	Caffeic acid [91]	GC [91]
			Cis- cinnamic acid [91]	
			Ferulic acid [91]	
			*p*-coumaric acid [91]	
	Shoots [83,90], root [90]	Phenolic acids	Hydrocaffeic acid [83]	CZE [90]; HPCE [83]
			Rosmarinic acid [83,90]	
			Chlorogenic acid [83]	
	Shoots [83], bee pollen [92]	Flavonoids	Kaempferol 3-glycoside [92]	HPCE [83]; TLC, 2D PC and HPLC [92]
			Quercetin 3-glycoside [92]	
			Rutin [83]	
	Root [82,83,90]	Naphthoquinones	Acetylshikonin [82]	CZE [90]; HPCE [83]; HPLC-UV [82]
			Deoxyshikonin [82]	
			Isovalerylshikonin [82]	
			Shikonin [82,83,90]	
			3,3-dimethylacrylshikonin [82]	
			2-methyl-*n*-butyrylshikonin [82]	
	Roots [93]	Sterone	Stigmast-4-ene-3,6-dione [93]	preparative TLC, ^1^H, 2D-NMR [93]
			β-sitosterol [93]	

RP-HPLC—reverse phase high-performance liquid chromatography, HPCE—high-performance capillary electrophoresis; UPLC-MS—ultra-performance liquid chromatography/mass spectrometry; TLC—thin layer chromatography; ^1^H-NMR—nuclear magnetic resonance; FAB-MS—fast atom bombardment/mass spectrometry; UV—ultraviolet; HPLC—high-performance liquid chromatography; HPLC-PAD-MS—high-performance liquid chromatography/photodiode-array detection coupled to ion trap mass spectrometry; DAD—diode array detection; ESI-MS—electrospray ionization/mass spectrometry; CZE—capillary zone electrophoresis; CC—column chromatography; UHPLC/Q-ToF MS—ultra-high-pressure liquid chromatography coupled to quadrupole time-of-flight mass spectrometry. See **S1** for an image of common antioxidants extracted from *Echium*.

## Supplementary Materials

The following are available online at https://www.mdpi.com/2076-3921/9/8/722/s1, Figure S1: A selection of pure compounds isolated from *Echium* spp. with potent antioxidant activity.

## References

[1] Baltisberger M., Widmer A. (2006). Chromosome numbers of plant species from the Canary Islands. Bot. Helv..

[2] Böhle U.-R., Hilger H.H., Martin W.F. (1996). Island colonization and evolution of the insular woody habit in *Echium* L.(Boraginaceae). Proc. Natl. Acad. Sci. USA.

[3] Mack R.N. (2003). Plant Naturalizations and Invasions in the Eastern United States: 1634-1860. Ann. Missouri Bot.

[4] Sayyah M., Boostani H., Pakseresht S., Malaieri A. (2009). Efficacy of aqueous extract of *Echium amoenum* in treatment of obsessive–compulsive disorder. Prog. NeuroPsychopharmacol. Biol. Psychiatry.

[5] Shafaghi B., Naderi N., Tahmasb L., Kamalinejad M. (2010). Anxiolytic effect of *Echium amoenum* L. in mice. Iran. J. Pharm. Sci..

[6] Rabbani M., Sajjadi S.E., Vaseghi G., Jafarian A. (2004). Anxiolytic effects of *Echium amoenum* on the elevated plus-maze model of anxiety in mice. Fitoterapia.

[7] Potdar V.H., Kibile S.J. (2011). Evaluation of Antidepressant-like Effect of *Citrus Maxima* Leaves in Animal Models of Depression. Iran J. Basic Med. Sci..

[8] Forcella F., Wood J., Dillon S. (1986). Characteristics distinguishing invasive weeds within *Echium* (Bugloss). Weed Res..

[9] Sharma G., Esler K. (2008). Phenotypic plasticity among *Echium plantagineum* populations in different habitats of Western Cape, South Africa. S. Afr. J. Bot.

[10] Konarzewski T.K., Murray B.R., Godfree R.C. (2012). Rapid development of adaptive, climate-driven clinal variation in seed mass in the invasive annual Forb *Echium plantagineum* L.. PLoS ONE.

[11] Zhu X., Skoneczny D., Weidenhamer J.D., Mwendwa J.M., Weston P.A., Gurr G.M., Callaway R.M., Weston L.A. (2016). Identification and localization of bioactive naphthoquinones in the roots and rhizosphere of Paterson’s curse (*Echium plantagineum*), a noxious invader. J. Exp. Bot..

[12] Heidari M.R., Azad E.M., Mehrabani M. (2006). Evaluation of the analgesic effect of *Echium amoenum* Fisch & C.A. Mey. extract in mice: Possible mechanism involved. J. Ethnopharmacol..

[13] Faryadian S., Sydmohammadi A., Khosravi A., Kashiri M., Faryadayn P., Abasi N. (2015). Aqueous Extract of *Echium amoenum* Elevate CSF Serotonin and Dopamine Level in Depression rat. Biomed. Pharmacol. J..

[14] Rabbani M., Sajjadi S.E., Khalili S. (2011). A Lack of tolerance to the anxiolytic action of *Echium amoenum*. Res. Pharm. Sci..

[15] Bekhradnia S., Ebrahimzadeh M.A. (2016). Antioxidant activity of *Echium amoenum*. Rev. Chim. J..

[16] Hosseini N., Abolhassani M. (2011). Immunomodulatory properties of borage (*Echium amoenum*) on BALB/c mice infected with Leishmania major. J. Clin. Immunol..

[17] Abolhassani M. (2004). Antibacterial effect of borage (*Echium amoenum*) on Staphylococcus aureus. Braz. J. Infect. Dis.

[18] Abolhassani M. (2010). Antiviral activity of borage (*Echium amoenum*). Arch. Med. Sci..

[19] Romeiras M.M., Paulo O.S., Duarte M.C., Pina-Martins F., Cotrim M.H., Carine M.A., Pais M.S. (2011). Origin and diversification of the genus *Echium* (Boraginaceae) in the Cape Verde archipelago. Taxon.

[20] El-Shazly A., Wink M. (2014). Diversity of pyrrolizidine alkaloids in the Boraginaceae structures, distribution, and biological properties. Diversity.

[21] Azizi H., Ghafari S., Ghods R., Shojaii A., Salmanian M., Ghafarzadeh J. (2018). A review study on pharmacological activities, chemical constituents, and traditional uses of *Echium amoenum*. Pharmacogn. Rev..

[22] Abbasi F., Jamei R. (2019). Effects of Silver Nanoparticles and Silver Nitrate on Antioxidant Responses in *Echium amoenum*. Russ. J. Plant Physiol..

[23] Ahvazi M., Khalighi-Sigaroodi F., Charkhchiyan M.M., Mojab F., Mozaffarian V.-A., Zakeri H. (2012). Introduction of medicinal plants species with the most traditional usage in Alamut region. Iran. J. Pharm. Res..

[24] Mehrabani M., Ghassemi N., Ghannadi E.S.A., Shams-Ardakani M. (2005). Main phenolic compound of petals of *Echium amoenum* Fisch. and CA Mey., a famous medicinal plant of Iran. DARU J. Pharm. Sci..

[25] Swamy M.K., Patra J.K., Rudramurthy G.R. (2019). Medicinal Plants: Chemistry, Pharmacology, and Therapeutic Applications.

[26] De Natale A., Pollio A. (2007). Plants species in the folk medicine of Montecorvino Rovella (inland Campania, Italy). J. Ethnopharmacol..

[27] Eruygur N., Yilmaz G., Kutsal O., Yücel G., Üstün O. (2016). Bioassay-guided isolation of wound healing active compounds from *Echium* species growing in Turkey. J. Ethnopharmacol..

[28] Sezik E., Yeşİlada E., Tabata M., Honda G., Takaishi Y., Fujita T., Tanaka T., Takeda Y. (1997). Traditional medicine in Turkey viii. folk medicine in east Anatolia; Erzurum, Erzíncan, Ağri, Kars, Iğdir provinces. Econ. Bot..

[29] Eruygur N., Yilmaz G., Üstün O. (2012). Analgesic and antioxidant activity of some *Echium* species wild growing in Turkey. Fabad J. Pharm. Sci..

[30] Foster S., Duke J.A. (1990). A field guide to medicinal plants: Eastern and central North America. The Peterson Field Guide Series (USA).

[31] Martín Arroyo T., González-Porto A.V., Bartolomé Esteban C. (2017). Viper’s bugloss (*Echium* spp.) honey typing and establishing the pollen threshold for monofloral honey. PLoS ONE.

[32] Klemow K.M., Clements D.R., Threadgill P.F., Cavers P.B. (2002). The biology of Canadian weeds. 116. Echium vulgare L. Can. J. Plant Sci..

[33] Félix-Silva J., Silva-Junior A.A., Zucolotto S.M., Fernandes-Pedrosa M.d.F. (2017). Medicinal plants for the treatment of local tissue damage induced by snake venoms: An overview from traditional use to pharmacological evidence. Evid. Based Complementary Altern. Med..

[34] Rigat M., Vallès J., Gras A., Iglésias J., Garnatje T. (2015). Plants with topical uses in the Ripollès district (Pyrenees, Catalonia, Iberian Peninsula): Ethnobotanical survey and pharmacological validation in the literature. J. Ethnopharmacol..

[35] Yeşilada E., Honda G., Sezik E., Tabata M., Fujita T., Tanaka T., Takeda Y., Takaishi Y. (1995). Traditional medicine in Turkey. V. Folk medicine in the inner Taurus Mountains. J. Ethnopharmacol..

[36] Fujita T., Sezik E., Tabata M., Yesilada E., Honda G., Takeda Y., Tanaka T., Takaishi Y. (1995). Traditional medicine in Turkey VII. Folk medicine in middle and west Black Sea regions. Econ. Bot..

[37] Alali F.Q., Tahboub Y.R., Ibrahim E.S., Qandil A.M., Tawaha K., Burgess J.P., Sy A., Nakanishi Y., Kroll D.J., Oberlies N.H. (2008). Pyrrolizidine alkaloids from *Echium glomeratum* (Boraginaceae). Phytochemistry.

[38] Olennikov D., Daironas Z.V., Zilfikarov I. (2017). Shikonin and Rosmarinic-Acid Derivatives from *Echium russicum* Roots. Chem. Nat. Compd..

[39] Altundag E., Ozturk M. (2011). Ethnomedicinal studies on the plant resources of east Anatolia, Turkey. Procedia Soc. Behav. Sci..

[40] Kuete V., Wiench B., Alsaid M.S., Alyahya M.A., Fankam A.G., Shahat A.A., Efferth T. (2013). Cytotoxicity, mode of action and antibacterial activities of selected Saudi Arabian medicinal plants. BMC Complement Altern. Med..

[41] Al-Hamaideh K.D., El-Elimat T., Afifi F.U., Kasabri V. (2017). Phytochemical screening and pharmacological activities of *Echium judaeum* lacaita extracts growing wild in Jordan. Jordan J. Pharm. Sci..

[42] González-Tejero M., Molero-Mesa J., Casares-Porcel M., Lirola M.M. (1995). New contributions to the ethnopharmacology of Spain. J. Ethnopharmacol..

[43] Abdel-Sattar E., Harraz F.M., Al-Ansari S.M., El-Mekkawy S., Ichino C., Kiyohara H., Otoguro K., Omura S., Yamada H. (2009). Antiplasmodial and antitrypanosomal activity of plants from the Kingdom of Saudi Arabia. J. Nat. Med..

[44] Lima I.S.M. (2013). Medicinal and Aromatic Plants of Cape Verde: Characterization of Volatile Metabolites of Endemic and Native Species. Ph.D. Thesis.

[45] Carvalho J.C.B., dos Santos Almeida H., Lobo J.F.R., Ferreira J.L.P., Oliveira A.P., Rocha L. (2013). Pyrrolizidine alkaloids in two endemic capeverdian *Echium* species. Biochem. Syst. Ecol..

[46] Ünsal Ç., Vural H., Sariyar G., Özbek B., Ötük G. (2010). Traditional medicine in Bilecik province (Turkey) and antimicrobial activities of selected species. Turk. J. Pharm. Sci..

[47] González-Tejero M., Casares-Porcel M., Sánchez-Rojas C., Ramiro-Gutiérrez J., Molero-Mesa J., Pieroni A., Giusti M., Censorii E., De Pasquale C., Della A. (2008). Medicinal plants in the Mediterranean area: Synthesis of the results of the project Rubia. J. Ethnopharmacol..

[48] Conforti F., Sosa S., Marrelli M., Menichini F., Statti G.A., Uzunov D., Tubaro A., Menichini F. (2009). The protective ability of Mediterranean dietary plants against the oxidative damage: The role of radical oxygen species in inflammation and the polyphenol, flavonoid and sterol contents. Food Chem..

[49] Abed A., Vaseghi G., Jafari E., Fattahian E., Babhadiashar N., Abed M. (2014). *Echium Amoenum* Fisch. Et Mey: A review on its pharmacological and medicinal properties. Asian J. Med. Pharm. Res..

[50] Pilerood S.A., Prakash J. (2014). Evaluation of nutritional composition and antioxidant activity of Borage (*Echium amoenum*) and Valerian (*Valerian officinalis*). Int. J. Food Sci. Technol..

[51] Noroozpour Dailami K., Azadbakht M., Lashgari M., Rashidi Z. (2015). Prevention of selenite-induced cataractogenesis by hydroalchoholic extract of *Echium amoenum*: An experimental evaluation of the Iranian traditional eye medication. Pharm. Biomed. Res..

[52] Asghari B., Mafakheri S., Zarrabi M., Erdem S., Orhan I., Bahadori M. (2019). Therapeutic target enzymes inhibitory potential, antioxidant activity, and rosmarinic acid content of *Echium amoenum*. S. Afr. J. Bot.

[53] Zhang Y.-J., Gan R.-Y., Li S., Zhou Y., Li A.-N., Xu D.-P., Li H.-B. (2015). Antioxidant phytochemicals for the prevention and treatment of chronic diseases. Molecules.

[54] Chaouche T.M., Haddouchi F., Ksouri R., Atik-Bekkara F. (2014). Evaluation of antioxidant activity of hydromethanolic extracts of some medicinal species from South Algeria. J. Chin. Med. Assoc..

[55] Radwan H., Shams K., Mohamed W., Ismail I. (2007). Phytochemical and biological investigation of *Echium sericeum* (Vahl) growing in Egypt. Planta Med..

[56] Dawidar A.M., Ghani A., Alshamy M., Tawfik E., Abdel-Mogib M. (2015). Fatty Acid Pattern and alkaloids of *Echium rauwolfii*. Int. J. Appl. Sci. Eng..

[57] Kefi S., Essid R., Mkadmini K., Kefi A., Haddada F.M., Tabbene O., Limam F. (2018). Phytochemical investigation and biological activities of *Echium arenarium* (Guss) extracts. Microb. Pathog..

[58] Nićiforović N., Mihailović V., Mašković P., Solujić S., Stojković A., Muratspahić D.P. (2010). Antioxidant activity of selected plant species; potential new sources of natural antioxidants. Food Chem. Toxicol..

[59] Nadi F. (2017). Bioactive compound retention in *Echium amoenum* Fisch. & CA Mey. petals: Effect of fluidized bed drying conditions. Int. J. Food Prop..

[60] Tahmouzi S. (2014). Extraction, antioxidant and antilisterial activities of polysaccharides from the flower of viper’s bugloss. Int. J. Biol. Macromol..

[61] Dresler S., Wójciak-Kosior M., Sowa I., Stanisławski G., Bany I., Wójcik M. (2017). Effect of short-term Zn/Pb or long-term multi-metal stress on physiological and morphological parameters of metallicolous and nonmetallicolous *Echium vulgare* L. populations. Plant Physiol. Biochem..

[62] Hashemi Z., Ebrahimzadeh M.A., Khalili M. (2019). Sun protection factor, total phenol, flavonoid contents and antioxidant activity of medicinal plants from Iran. Trop. J. Pharm. Res..

[63] Abbaszadeh S., Radjabian T., Taghizadeh M. (2013). Antioxidant Activity, Phenolic and Flavonoid Contents of *Echium* Species from Different Geographical Locations of Iran. J. Med. Plants By-Prod..

[64] Tenuta M.C., Tundis R., Xiao J., Loizzo M.R., Dugay A., Deguin B. (2019). *Arbutus species* (Ericaceae) as source of valuable bioactive products. Crit. Rev. Food Sci. Nutr..

[65] Vukajlović F.N., Pešić S.B., Tanasković S.T., Predojević D.Z., Gvozdenac S.M., Prvulović D.M., Bursić V.P. (2018). Efficacy of *Echium spp*. water extracts as post-harvest grain protectants against Plodia interpunctella. Rom. Biotechnol. Lett..

[66] Birben E., Sahiner U.M., Sackesen C., Erzurum S., Kalayci O. (2012). Oxidative stress and antioxidant defense. World Allergy Organ. J..

[67] Safaeian L., Javanmard S.H., Ghanadian M., Seifabadi S. (2015). Cytoprotective and antioxidant effects of *Echium amoenum* anthocyanin-rich extract in human endothelial cells (HUVECs). Avicenna J. Phytomed..

[68] Abbasi Larki R., Zayerzadeh E., Harzandi N., Anissian A. (2020). Protective Effects of *Echium amoenum* on Oxidative Stress and Gene Expression Induced by Permethrin in Wistar Rats. Hepat Mon..

[69] Abed A., Minaiyan M., Ghannadi A., Mahzouni P., Babavalian M.R. (2012). Effect of *Echium amoenum* Fisch. et Mey a traditional Iranian herbal remedy in an experimental model of acute pancreatitis. ISRN Gastroenterol..

[70] Kamali M., Johari H. (2017). The antioxidant effect of *Echium amoenum* to prevent teratogenic effects of Lamotrigine on the skeletal system and fetal growth in mice. J. Pharmacol. Clin. Res..

[71] Ranjbar A., Khorami S., Safarabadi M., Shahmoradi A., Malekirad A.A., Vakilian K., Mandegary A., Abdollahi M. (2006). Antioxidant activity of Iranian *Echium amoenum* Fisch & CA Mey flower decoction in humans: A cross-sectional before/after clinical trial. Evid. Based Complementary Altern. Med..

[72] Dai J., Mumper R.J. (2010). Plant phenolics: Extraction, analysis and their antioxidant and anticancer properties. Molecules.

[73] Pandey K.B., Rizvi S.I. (2009). Plant polyphenols as dietary antioxidants in human health and disease. Oxid. Med. Cell. Longev..

[74] Fazly Bazzaz B., Haririzadeh G., Imami S., Rashed M. (1997). Survey of Iranian plants for alkaloids, flavonoids, saponins, and tannins [Khorasan Province]. Int. J. pharmacogn..

[75] Chaouche T., Haddouchi F., Bekkara F.A. (2011). Phytochemical study of roots and leaves of the plant *Echium pycnanthum* Pomel. Der Pharm. Lett..

[76] Sousa C., Moita E., Valentão P., Fernandes F., Monteiro P., Andrade P.B. (2015). Effects of colored and noncolored phenolics of *Echium plantagineum* L. bee pollen in Caco-2 cells under oxidative stress induced by tert-butyl hydroperoxide. J. Agric. Food Chem..

[77] Panche A., Diwan A., Chandra S. (2016). Flavonoids: An overview. J. Nutr. Sci..

[78] Di Paola-Naranjo R.D., Sánchez-Sánchez J., González-Paramás A.M., Rivas-Gonzalo J.C. (2004). Liquid chromatographic–mass spectrometric analysis of anthocyanin composition of dark blue bee pollen from *Echium plantagineum*. J. Chromatogr. A.

[79] Moita E., Gil-Izquierdo A., Sousa C., Ferreres F., Silva L.R., Valentão P., Domínguez-Perles R., Baenas N., Andrade P.B. (2013). Integrated Analysis of COX-2 and iNOS Derived Inflammatory Mediators in LPS-Stimulated RAW Macrophages Pre-Exposed to *Echium plantagineum* L. Bee Pollen Extract. PLoS ONE.

[80] Ferreres F., Pereira D.M., Valentão P., Andrade P.B. (2010). First report of non-coloured flavonoids in *Echium plantagineum* bee pollen: Differentiation of isomers by liquid chromatography/ion trap mass spectrometry. Rapid Commun. Mass Spectrom..

[81] Durán A.G., Gutiérrez M.T., Rial C., Torres A., Varela R.M., Valdivia M.M., Molinillo J.M., Skoneczny D., Weston L.A., Macías F.A. (2017). Bioactivity and quantitative analysis of isohexenylnaphthazarins in root periderm of two *Echium spp*.: *E. plantagineum* and *E. gaditanum*. Phytochemistry.

[82] Eruygur N. (2018). A simple isocratic high-perfomance liquid chromatography method for the simultaneous determination of shikonin derivatives in some *Echium* species growing wild in Turkey. Turk. J. Pharm. Sci..

[83] Dresler S., Szymczak G., Wójcik M. (2017). Comparison of some secondary metabolite content in the seventeen species of the boraginaceae family. Pharm. Biol..

[84] Albreht A., Vovk I., Simonovska B., Srbinoska M. (2009). Identification of shikonin and its ester derivatives from the roots of *Echium italicum* L.. J. Chromatogr. A.

[85] Zare K., Khosrowshahli M., Nazemiyeh H., Movafeghi A., Azar A.M., Omidi Y. (2011). Callus culture of *Echium italicum* L. towards production of a shikonin derivative. Nat. Prod. Res..

[86] Weston P.A., Weston L.A., Hildebrand S. (2013). Metabolic profiling in *Echium plantagineum*: Presence of bioactive pyrrolizidine alkaloids and napthoquinones from accessions across southeastern Australia. Phytochem. Rev..

[87] Skoneczny D., Zhu X., Weston P.A., Gurr G.M., Callaway R.M., Weston L.A. (2019). Production of pyrrolizidine alkaloids and shikonins in *Echium plantagineum* L. in response to various plant stressors. Pest Manag. Sci..

[88] Fukui H., Tsukada M., Mizukami H., Tabata M. (1983). Formation of stereoisomeric mixtures of naphthoquinone derivatives in *Echium lycopsis* callus cultures. Phytochemistry.

[89] Chaouche T., Haddouchi F., Bekkara F.A. (2012). Identification of shikonin from the roots of *Echium pycnanthum* Pomel. Asian J. Pharm. Clin. Res..

[90] Dresler S., Kubrak T., Bogucka-Kocka A., Szymczak G. (2015). Determination of shikonin and rosmarinic acid in *Echium vulgare* L. and *Echium russicum* JF Gmel. by capillary electrophoresis. J. Liq. Chromatogr. Relat..

[91] Mitkov S., Obreshkova D., Ilieva I., Pangarova T., Pencheva I. (2002). Phenolcarboxylic acids in *Echium vulgare* L. Acta Pharma. Turcica.

[92] Tomás-Barberán F.A., Tomás-Lorente F., Ferreres F., Garcia-Viguera C. (1989). Flavonoids as biochemical markers of the plant origin of bee pollen. J. Sci. Food Agric..

[93] Pardo F., Perich F., Torres R., Delle Monache F. (2000). Stigmast-4-ene-3, 6-dione an unusual phytotoxic sterone from the roots of *Echium vulgare* L.. Biochem. Syst. Ecol..

[94] Dresler S., Rutkowska E., Bednarek W., Stanisławski G., Kubrak T., Bogucka-Kocka A., Wójcik M. (2017). Selected secondary metabolites in Echium *vulgare* L. populations from nonmetalliferous and metalliferous areas. Phytochemistry.

[95] Papageorgiou V.P., Assimopoulou A.N., Couladouros E.A., Hepworth D., Nicolaou K. (1999). The chemistry and biology of alkannin, shikonin, and related naphthazarin natural products. Angew. Chem. Int. Ed..

[96] Chen X., Yang L., Oppenheim J.J., Howard O.Z. (2002). Cellular pharmacology studies of shikonin derivatives. Phytother. Res..

[97] Paniagua-Pérez R., Madrigal-Bujaidar E., Reyes-Cadena S., Alvarez-González I., Sánchez-Chapul L., Pérez-Gallaga J., Hernández N., Flores-Mondragón G., Velasco O. (2008). Cell protection induced by beta-sitosterol: Inhibition of genotoxic damage, stimulation of lymphocyte production, and determination of its antioxidant capacity. Arch. Toxicol..

[98] Gupta R., Sharma A.K., Dobhal M., Sharma M., Gupta R. (2011). Antidiabetic and antioxidant potential of β-sitosterol in streptozotocin-induced experimental hyperglycemia. J. Diabetes.

