# Supplementary figures and images for: Antioxidant Properties and Reported Ethnomedicinal Use of the Genus Echium (Boraginaceae)
Source: Antioxidants (Basel). 2020 Aug 9;9(8):722. doi: 10.3390/antiox9080722 (PMC7466025; doi:10.3390/antiox9080722)

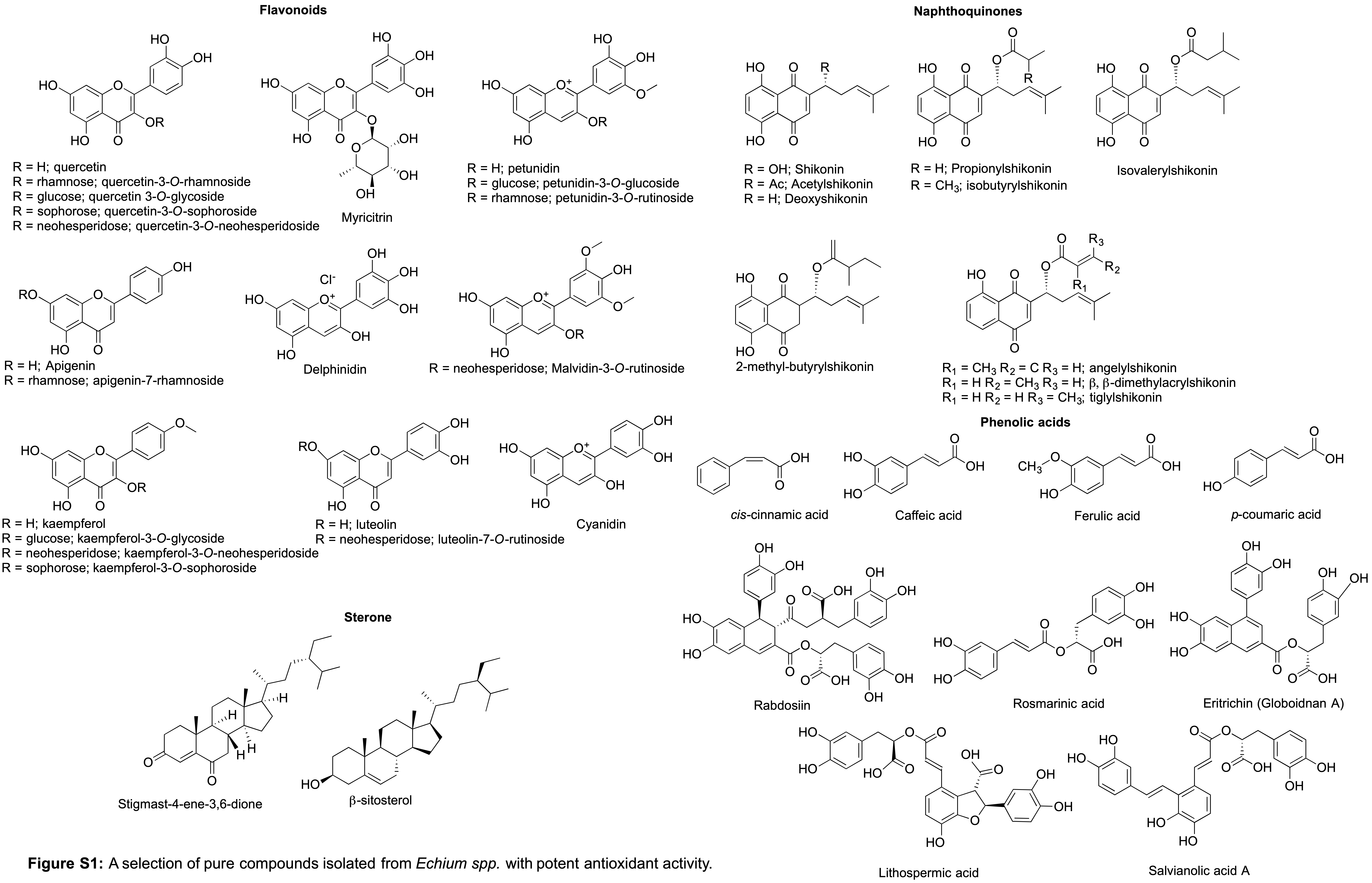

Supplement: Supplementary file 1 [file antioxidants-09-00722-s001.zip › antioxidants-865804-supplementary.png]
